# The voice of patients in system redesign: A case study of redesigning a centralized system for intake of referrals from primary care to rheumatologists for patients with suspected rheumatoid arthritis

**DOI:** 10.1111/hex.12855

**Published:** 2018-12-05

**Authors:** Elena Lopatina, Jean L. Miller, Sylvia R. Teare, Nancy J. Marlett, Jatin Patel, Claire E. H. Barber, Dianne P. Mosher, Tracy Wasylak, Linda J. Woodhouse, Deborah A. Marshall

**Affiliations:** ^1^ Department of Community Health Sciences Cumming School of Medicine University of Calgary Calgary Alberta Canada; ^2^ O'Brien Institute for Public Health University of Calgary Calgary Alberta Canada; ^3^ Community Rehabilitation and Disability Studies Department of Community Health Sciences Cumming School of Medicine University of Calgary Calgary Alberta Canada; ^4^ Strategic Clinical Networks™ Alberta Health Services Edmonton Alberta Canada; ^5^ Department of Medicine Cumming School of Medicine University of Calgary Calgary Alberta Canada; ^6^ Arthritis Research Canada Vancouver British Columbia Canada; ^7^ Faculty of Nursing University of Calgary Calgary Alberta Canada; ^8^ Department of Physical Therapy Faculty of Rehabilitation Medicine University of Alberta Edmonton Alberta Canada; ^9^ McCaig Institute for Bone and Joint Health Calgary Alberta Canada; ^10^ Alberta Bone and Joint Health Institute Calgary Alberta Canada

**Keywords:** health system redesign, patient engagement in research and system redesign, patient needs, patient‐centred care, patient‐to‐patient research

## Abstract

**Background:**

The published literature demands examples of health‐care systems designed with the active engagement of patients to explore the application of this complex phenomenon in practice.

**Methods:**

This case study explored how the voice of patients was incorporated into the process of redesigning an element of the health‐care system, a centralized system for intake of referrals from primary care to rheumatologists for patients with suspected rheumatoid arthritis (RA)—centralized intake. The phenomenon of patient engagement using “patient and community engagement researchers” (PaCERs) in research and the process of redesigning centralized intake were selected as the case. In‐depth evaluation of the case was undertaken through the triangulation of findings from the document review and participants’ reflection on the case.

**Results:**

In this case, patients and PaCERs participated in multiple activities including an initial meeting of key stakeholders to develop the project vision; a patient‐to‐patient PaCERs study to gather perspectives of patients with RA on the challenges they face in accessing and navigating the health‐care system, and what they see as key elements of an effective system that would be responsive to their needs; the development of an evaluation framework for future centralized intake; and the choice of candidate centralized intake strategies to be evaluated.

**Conclusions:**

The described feasible multistep approach to active patient engagement in health‐care system redesign contributes to an understanding of the application of this complex phenomenon in practice. Therefore, the manuscript serves as one more step towards a patient‐centred health‐care system that is redesigned with active patient engagement.

## 1 INTRODUCTION

During the last decades, health‐care organizations around the world have been actively advocating for patient‐centred care as means of ensuring high‐quality care.^1^, ^2^, ^3^, ^4^ Patient‐centred care calls for a more holistic approach to care delivery that is focused on patients’ needs and experiences of well‐being and illness from a multidimensional biopsychosocial perspective.^5^, ^6^, ^7^, ^8^ To achieve patient‐centred care, fundamental changes, including a redesign of existing systems and/or design of new ones, are required.^9^

Integrating patients (ie, patient involvement^10^) into research and system design has been recognized as important elements in achieving patient‐centred care.^11^ Patient involvement offers the potential to target research and system design to patients’ needs, thus improving the patient experience with care and quality of care, and potentially reduce costs of care.^12^, ^13^, ^14^ Despite decades of discussions about the importance and potential benefits of patient involvement in health planning, research and system design, to date, patient involvement in designing and redesigning health‐care systems has often been limited to passive involvement.^10^, ^15^, ^16^ Few examples where patients and other stakeholders have been actively engaged as partners to design and redesign the system are available in the literature.^12^, ^17^ Therefore, more examples of active engagement of patients and other stakeholders in the system redesign are needed to explore the application of this complex phenomenon in practice.^10^, ^15^, ^16^, ^18^

This manuscript reports on a case study of patient involvement in redesigning a centralized system for intake of referrals from primary care to rheumatologists for patients with suspected rheumatoid arthritis (RA), hereafter referred to as centralized intake (CI).

## 2 METHODS

**Figure 1:**
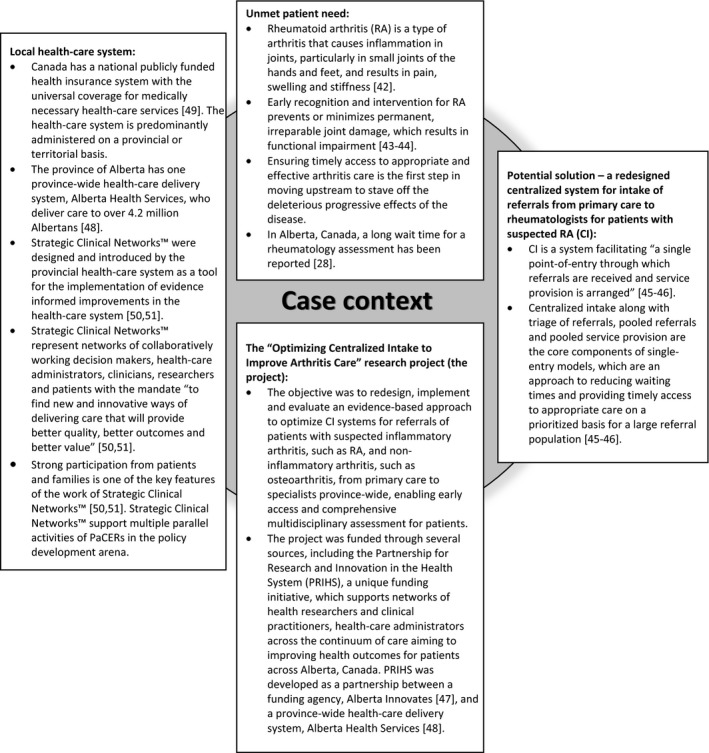
Environment and context for the case of patient and PaCERs engagement in the “Optimizing Centralized Intake to Improve Arthritis Care” project. Sources: Badley,^42^ Goekoop‐Ruiterman et al,^43^ Pope et al,^44^ Hazlewood et al,^28^ Damani et al,^45^ Lopatina et al,^46^ Alberta Innovates,^47^ Alberta Health Services,^48^ Government of Canada,^49^ Alberta Health Services^50^ and Noseworthy et al^51^

The study followed the case study design approach as described by Yin.^19^ In this study, the phenomenon of patient engagement using “patient and community engagement researchers” (PaCERs) in research and the process of redesigning CI were selected as the case.^19^ PaCERs are citizens living with various health conditions who received formal research training that includes how to design research, engage other patients and conduct research projects using an established protocol of qualitative inquiry.^20^, ^21^, ^22^ The case took place within the unique context of a 2‐year‐long research project, which aimed at “Optimizing Centralized Intake to Improve Arthritis Care,” hereafter referred as the project (Figure 1). The case study aimed to explore how the voice of patients was incorporated into the process of redesigning an element of the health‐care system, CI, within the project. The case study research team consisted of multiple stakeholders (PaCERs, academic researchers, health‐care professionals and health‐care administrators), who were engaged in the project. The study was driven by two predefined theoretical propositions^19^: (a) health‐care systems should be responsive to patients’ needs,^2^ and (b) active patient engagement (ie, patient involvement^10^) in system design and redesign is required to build a system that is responsive to patient needs.^11^ A detailed reporting on each activity within the project (eg, objectives, participants, actions, decisions and results) was undertaken by the project manager (JP) and academic researcher leading the project (DAM) to facilitate the case study. All documents that reported on the case and the context of the case were reviewed by the project manager (JP) and two academic researchers (DAM, EL) with expertise in qualitative and mixed‐methods research to extract data on objectives of patient and PaCERs engagement in redesigning CI, roles of patients and PaCERs in the project, and outcomes of their engagement. An inductive narrative analysis of the extracted data was conducted by three academic researchers (DAM, EL, NM) with expertise in qualitative and mixed‐methods research and two PaCERs (JLM, SRT) to summarize the in‐depth description of the case and preliminary findings of the case study.^19^, ^23^ Next, other members of the team reflected on the preliminary findings and refined them from the perspective of their personal experience with participating in the project and observation of the case. Lastly, preliminary findings from the document review and the team's reflection were triangulated through team discussions to generate the final findings.

The research was approved by the University of Calgary Conjoint Health Research Ethics Board (Ethics ID number REB13‐0822).

## 3 RESULTS

Patient and community engagement researchers were engaged in the project from the outset as equal partners with the rest of the project team members. This involved setting up the research agenda, helping to develop the funding application and applying for funding. During the project execution, patients and PaCERs participated in multiple activities described in detail here below and presented in chronological order.

### 3.1 First stakeholder meeting

At the beginning of the project execution, the project team engaged key stakeholders (patients and PaCERs [n = 4], health‐care professionals [n = 11], health‐care administrators [n = 15] and academic researchers [n = 8]) for a 1‐day meeting to develop the project vision. The meeting started with a discussion about centralized systems for intake of referrals as a construct, including a set of definitions to establish a common language. Subsequently, attendees were engaged in a facilitated discussion of three questions: (a) Can centralized systems for intake of referrals facilitate optimal care for patients living with RA? (b) How can essential services for patients living with RA be integrated in a continuous pathway? (c) How can quality of care be measured in a meaningful manner that the findings can influence patient outcomes and health system efficiency? Throughout the discussion, a scribe took notes. Meeting notes were summarized using thematic analysis by the research team (DAM, JP) to identify common themes. Next, meeting notes and identified themes were circulated to all meeting participants to be checked for consistency and reviewed for comments. Afterwards, the research team (DAM, JP) incorporated participants’ comments and refined the identified themes. Based on those themes, the project vision was framed as a set of principles aimed at developing an optimal CI, which should:

1. Create a high‐quality experience with the process for patients and providers;
2. Ensure patients’ timely access to the appropriate care pathway;
3. Ensure patients engage (and are engaged by) the appropriate care providers with a minimal number of referrals from one specialist to another;
4. Ensure patients are triaged and referred to appropriate care providers based on “best practice” to achieve desired outcomes;
5. Ensure resources are optimally used in achieving desired outcomes;
6. Mitigate risks to avoid unintended or harmful results.

Once established, these targets shaped and guided subsequent project phases. In particular, the patient‐centred nature of the majority of these principles highlighted the demand for further input from patients into the processes of redesign, implementation and evaluation of CI to ensure that the future system is responsive to patients’ needs.

### 3.2 PaCERs study

Next, PaCERs conducted a study to gather perspectives of patients with RA on the challenges they face in accessing and navigating the health‐care system, and what they see as key elements of an effective system that would be responsive to their needs. Although the focus of this work was on CI, the PaCERs study explored patients’ perspectives on the entire care pathway. This was done to account for the complexity of the health‐care system and possible interactions between the system's elements, as CI is just one element of the care pathway for patients. The study was led by two PaCERs who are patients living with osteoarthritis with previous experience in research on care delivery for patients with musculoskeletal conditions (JLM, SRT) and a research assistant who is a patient living with RA. Participants who self‐identified as having RA were recruited through arthritis networks, posters in rheumatology clinics and rheumatologists’ referrals. Those interested in the study contacted PaCERs, who sent potential participants the study description and a consent form. Next, completed consent forms were sent back to PaCERs. The PaCERs study went through three phases: *set*,* collect* and *reflect* as outlined in the PaCERs methodology.^20^, ^21^ Given the iterative nature of the research, patients were recruited and data were collected until data saturation was reached at each phase of the study. Over the three phases, 15 patients were included (Table 1).

**Table 1:** Self‐reported characteristics of participants (n = 15) in the patient and community engagement researchers (PaCERs) study

Characteristic	Number (percentage) of participants
Female	13 (87%)
Age groups	
Less than 40 y old	3 (20%)
40‐60 y old	5 (33%)
Over 60 y old	7 (47%)
Living in a large urban centre	11 (73%)
Newly diagnosed RA (approximately a year ago)	4 (27%)

#### 3.2.1 Set phase

Patient and community engagement researchers developed a focus group interview guide for the *set* phase based on the proceedings of the stakeholder meeting. The *set* focus group took place at neutral grounds for all attendees and lasted about 4 hours. During the *set* focus group, participants (n = 4) were first asked to talk about their experience being a patient with RA. Then, participants moved into a discussion about their experiences interacting with the health‐care system to manage their RA. The focus group ended with participants giving advice on what to address in the collect interviews. This included such factors as getting diagnosed, accessing a rheumatologist when medications need adjusting, communication between primary care providers and other providers, and maintaining patients’ mental well‐being. Throughout the focus group, participants’ points were documented on flip charts. Subsequently, PaCERs used focus group notes to develop a semistructured interview guide for the collect phase.

#### 3.2.2 Collect phase

In telephone interviews (n = 11), participants were asked about their experience of managing RA and their medications; what or who was most helpful; what other help they could use; and what was key to a system that would be responsive to the needs of patients with RA. Interviews lasted between 1 and 2 hours. Interviews were audiotaped, and notes were taken to document key points. Subsequently, PaCERs independently reviewed notes and the audiotape of each interview for salient points about access and navigating the health‐care system and key elements of a system that would be responsive to the needs of patients with RA and coded them using the grounded theory method and open coding technique.^24^ PaCERs then compared and contrasted their findings and, using a collaborative, iterative process, identified a preliminary set of themes and subthemes. PaCERs also identified challenges that patients faced when accessing and navigating the system and key elements of a system that patients reported would be responsive to patients’ needs. Next, preliminary findings were compiled and presented for discussion during the reflect focus groups.

#### 3.2.3 Reflect phase

There were three *reflect* focus groups that included a total of 10 participants: two for participants living in urban areas and one for participants from rural areas. The *reflect* focus groups took place locations neutral and convenient for all attending (eg, meeting room at a church or community centre) and lasted between 3 and 5 hours. PaCERs presented preliminary findings of the *collect* interviews. Then, PaCERs asked the focus group participants to reflect on the extent to which each of the identified challenges and key elements of the system that would be responsive to patients’ needs fit with what had previously been discussed during the collect phase. This was followed by a discussion on centralized systems for intake of referrals and the potential role of CI in addressing the key elements of the system that would be responsive to patients’ needs. The points discussed were documented on flip charts. Through a collaborative, iterative process, PaCERs examined the reflect focus group data to refine the preliminary findings and to develop the final findings.^24^ As a result, five themes were identified: (a) initial access to rheumatology care; (b) on‐going access to rheumatology care; (c) information about RA and resources for those living with RA; (d) fear of the future; and (e) collaborative and continuous care (Table 2). The final set of challenges that patients face when accessing and navigating the system, and key elements of the system that would be responsive to patients’ needs, were mapped to the corresponding themes (Table 3).

**Table 2:** Themes and subthemes identified through the patient and community engagement researchers (PaCERs) study with the corresponding examples of participants’ experience in accessing and navigating the health‐care system for management of rheumatoid arthritis (RA)

Themes	Subthemes	Examples of participants’ experience when accessing and navigating the health‐care system for management of RA
1. Initial access to rheumatology care	Delay in recognition of RA and the referral of patients to rheumatology care team by family doctors	All participants sought first help with joint pain from their primary care providers, which was a challenging experience for some of them For one participant, it took 2 y and multiple visits to her family doctor to get diagnosed, as her doctor did not believe she was in as much pain as she claimed. After she finally changed doctors, she was referred to a rheumatologist and diagnosed with RA within a week The family doctor of a young mother who could not pick up her baby due to pain suggested her pain might be related to her being depressed Stressing the importance of early diagnosis, one interviewee suggested family doctors should be more decisive in figuring out what is wrong, avoiding a “by guess and by golly” approach While positive rheumatoid factor test results expedited referral to a rheumatologist, negative results (that in the end turned out to be a false negative) delayed referral. After 4 mo of pain, one participant with a negative test finally went to the emergency department where she was admitted to hospital and diagnosed with RA
	Long waiting time for initial appointment	For one participant, it took 10 mo to get an appointment with the rheumatologist after her family doctor's referral Knowing it can take time to get a rheumatologist appointment, some patients’ family doctors started them on RA medications, for example a low dose of methotrexate. While the resulting pain control was a great relief, for some patients this led to further diagnosis delays since rheumatologists had to wait for the medication to clear their systems
	Positive experience with the initial access to rheumatology care	One participant described her experience of getting diagnosed as “a positively deviant case.” Participant's symptoms developed suddenly with painful swelling in most joints. She went to her family doctor's clinic, was sent for blood work and x‐rays right away and started on medication to manage the inflammation and pain while waiting for results. The test results indicated inflammation, and her family doctor got a telephone consult with a rheumatologist. This was on a Friday. The rheumatologist said she would see her Monday morning and suggested that if she could stand the pain over the weekend, she not start on steroids, which would mask her symptoms, as she and her doctor had planned. The participant agreed to this. On Monday, she was examined by a rheumatologist and diagnosed with RA. On Tuesday, she attended a class on RA medications taught by a pharmacist, and by Wednesday, the swelling and pain were under control. By Thursday, she had a follow‐up education session with the clinic nurse and started her medications. Since then, she has been followed regularly by both her family doctor and her rheumatologist and has good access to clinic staff if questions concerning treatment arise
	Suggestions for improving patients’ experience with the initial access to rheumatology care	Participants thought that awareness of family doctors of RA could be higher Participants believed that increased awareness of family doctors of RA would facilitate earlier referral to rheumatologists During the *reflection* phase, the participant, who had a positive experience in the initial access to rheumatology care, thought this “positively deviant experience” happened for a number of reasons. First, she recognized something fairly significant was happening to her, and when symptoms persisted, she made an appointment at her family practice clinic. Second, her family doctor managed her care well—she had been his patient for a long time and knew she “didn't come there lightly.” Third, on the follow‐up appointment about her first blood test results her doctor phoned the rheumatologist on call and together they started to figure out what to do next. In addition, as a patient she is comfortable in navigating the health‐care system, is not afraid to ask questions and is a strong believer in involving others in care decisions
2. On‐going access to rheumatology care	Challenges in accessing rheumatologists in case of flare or problems with medications	All participants were aware of the need for on‐going monitoring of their disease activity and medical management One participant who was on her third biologic said: “all of a sudden it was a wonder drug, and as quickly as it started to work it stopped” All participants mentioned they constantly worried that RA might flare up or medications might stop working. Patients worried that they would not be able to access the specialty clinic in case of such emergency
	Direct contact to rheumatology care team	Some participants reported to have direct telephone numbers or email addresses for the rheumatologists or nurses from the rheumatology team One participant thought being able to talk to a nurse practitioner is “brilliant, wonderful” as it provided patients access to the resources they need to keep their disease in control One participant had access to the pharmacist whose class she attended, describing this person as being “amazingly available,” responding to emails within hours: “I feel I have access, I don't have to wait for my 3‐month appointment to get access to the people who will answer my questions” In one situation, no one at the rheumatologist's office answered the phone so the participant had to go to emergency several times in 3 mo where she saw a rheumatologist on call
	Suggestions for improving patients’ experience with the on‐going access to rheumatology care	Having a direct contact with professionals with RA expertise was the preferred option for the participants to ensure on‐going access to care
3. Information about RA and resources for those living with RA	Lack of information about RA and resources for those living with RA	Participants reported receiving little if any information about RA when they were first diagnosed One patient described it as: “no nurse, no doctor, no physio, nobody has given me anything unless I find it myself” One participant from a remote community said she was “really in the dark” because she received no information from her rheumatologist, and there were no resources in her community Another participant described this as being left “floating in a big ocean all by myself with only a small bit of foam to stay afloat”
	Patients’ education is a professional responsibility of health‐care providers	One participant said that all newly diagnosed patients should receive a “whole package of education materials, and proper websites.” She believed that once a patient was diagnosed “a bunch of doors should start opening for you”; that patients you should be referred to physiotherapy and should be “pushed to go to certain classes” When some focus group participants mentioned had attended a medication class at one of the clinics they attended, the rest of participants started to wonder why their rheumatologists had not told them about such classes. As such, most participants concluded that RA specialists should be more aware of available resources
	Positive experience with education sessions for patients with RA	One participant said education sessions for people with RA taught her “how to manage pretty much any situation that can arise”
	Need for peer support and lack of peer support programs for those living with RA	Participants spoke of having few people to talk to about their RA and the need for peer support Participants described hiding their RA from their friends, family and coworkers because they did not want to be seen as different One participant said she told her friends that her bent finger was from a sports injury Participants shared that they often held back on developing relationships and one participant said that as the disease progresses she “shuts the world out” One person said that at one time she had been addicted to pain medications so now she would not talk to her husband about her pain as he worried she would get addicted again Those who are in the workforce did not talk about their RA with their employers and colleagues, as they did not want to be passed up for promotions or potentially lose their jobs Participants appreciated the opportunity the research gave them to meet and talk with others with similar worries
	Suggestions for improving patients’ experience with the access to information about RA and resources for those living with RA	Participants suggested that issues in accessing information and resources could be addressed through professionals’ active engagement in patients’ education about RA; comprehensive packages of information and resources on the disease, sequence of treatments and medications for newly diagnosed patients; and peer support resources In particular, participants highlighted the need for educational opportunities available in rural and smaller communities
4. Fear of the future	Fear of unknown	Participants worried about their future with respect to RA management, particularly, medications use Participants also worried about what would happen when they exhaust all available medications, as they knew that their current medications likely would not be effective forever One participant was told she was “almost at the end of her rope,” which she interpreted to mean there were not many other medications left for her to try For another patient, the only medication that worked was prednisone, and even though doctors caution her against long‐term side‐effects, she was reluctant to stop taking them: “sometimes I think I would just like to take the prednisone and possibly die early”
	Biologic drugs	Participants had a particular interest in biologics, which they considered to be the ultimate medications Some patients who were not on biologics wondered why they were not and pushed their doctors to prescribe biologics, while others hoped that they would not need to use them because of what they heard about the side‐effects
	Suggestions for reducing patients fear of the future	Participants wanted to be in charge of their own health Participants wanted to know and to understand the full range of medications used for RA Participants suggested that all patients should be educated on their current medications, other medications available and sequencing of those medications in order to prevent patients’ anxiety regarding their future
5. Collaborative and continuous care	Lack of collaborative and continuous care	Study participants described many instances where communication among family doctors and rheumatology care providers was lacking, which had negative impact on participants’ health Those with more than one health problem said there should be better communication between care providers involved in their care An older patient who had a heart problem and diabetes as well as RA was followed up by a family doctor, a cardiologist, an endocrinologist and a rheumatologist. In her experience, each specialist is only willing to deal with his or her own area, rather than treat her as a complex patient with multiple comorbidities. She thought that all specialists involved in her care were good, but because they did not communicate with each other, she was “lost in the shuffle”
	Suggestions for improving continuity of care	Participants concluded that patients need to be confident that their family doctors, specialists and RA professionals (rheumatologists, advanced practice nurses and pharmacists) communicate with each other Participants suggested that electronic records accessible to all their care providers could facilitate improved communication between health‐care providers and continuity of care

**Table 3:** Key elements of the health‐care system that would be responsive to patients’ needs aligned with corresponding themes identified through the patient and community engagement researchers (PaCERs) study

Themes identified during the PaCERs study	Summary of the issues faced by patients with RA in accessing and navigating the health‐care system	Key elements of the health‐care system that would be responsive to patients’ needs
1. Initial access to rheumatology care	Delay in recognition of RA by family doctors Delay in referral of patients to rheumatologists by primary care providers Long waiting time for initial appointment	Family doctors recognize the possibility of RA and refer patients to rheumatologists in a timely manner^a^ Effective mechanisms to facilitate communication between family doctors and rheumatologists at the point of referral are available^a^ Communication and collaboration between primary care providers, rheumatology team and the patient continues on the on‐going basis while waiting for the referral and after the initial appointment with the rheumatologist^a^
2. On‐going access to rheumatology care	Challenges in accessing rheumatologists in case of flare or problems with medications	On‐going access to the appropriate care provider (eg, rheumatologists, advanced practice nurses or pharmacists with RA expertise) is provided in a timely manner Patients have direct contact with a care provider specialized in rheumatology^a^
3. Information about RA and resources for those living with RA	Lack of educational programs and resources for those living with RA Lack of peer support programs Challenges in accessing educational programs and peer support programs for the patients living in rural areas	Multiple opportunities for patient education are provided^a^ Newly diagnosed patients receive a comprehensive package of information and resources on the disease, sequence of treatments, medications and peer support resources^a^ Referral to accessible education programs is provided during the initial access to rheumatology care^a^ Professionals actively engage patients in learning about RA^a^ Learning opportunities are available to patients in rural and smaller communities as well as urban centres^a^
4. Fear of the future	Patients have anxiety about available medication options and what would happen when they exhaust all available medications	Patients know the sequence of treatments for RA Patients understand the medications they are taking^a^ Patients understand what medications they may need in the future Patients have information on when biologics are used
5. Collaborative and continuous care	Lack of communication, connections and collaboration between family physician and rheumatology care providers Lack of communication, connections and collaboration between rheumatology care providers and other specialists involved in the patient care	Patients are confident their family doctors, specialists and RA professionals (rheumatologists, advanced practice nurses and pharmacists) communicate with each other and the patient on the on‐going basis Different specialists involved in the patient care communicate and collaborate to coordinate care provided Electronic records are used for communication and collaboration

a Key elements of a health‐care system that would be responsive to patients’ needs, which participants of the PaCERs study thought could be addressed to some extent through centralized intake.

#### 3.2.4 Interpretation of findings

Findings of the PaCERs study suggest that patient‐centred care for patients with RA should be viewed as a continuum. That continuum starts from the patients’ first point of contact with the health‐care system, carries through their initial appointment with a specialist and continues throughout their long‐term and on‐going follow‐up visits with their rheumatology care team. To be responsive to patients’ needs, the continuum of care should be easy to access and navigate. It should also provide patients easy access to information, education and community resources; as well as incorporate a communication infrastructure to promote collaboration among care providers. Participants reported experiencing multiple challenges when accessing and navigating the system. Among those challenges, initial access and on‐going access to care were raised as the two challenges that were of primary concern to participants.

The main goal of CI, as one element of the care pathway for patients with RA, is to ensure timely initial access to appropriate care. As such, participants found that CI had a potential to vastly improve patients’ experiences with care, as well as their outcomes, and should be considered when redesigning the system to be responsive to the needs of patients. Unfortunately, several participants experienced challenges with the initial access to specialists’ care due to delayed recognition of suspected RA by primary care providers. Therefore, patients recommended that CI should facilitate access to information and resources for primary care providers and patients to improve their knowledge about RA. CI could also play a role in improving communication and collaboration between primary care providers, the rheumatology care team and the patient through electronic communication methods.

Although coordination of on‐going follow‐up is not among the goals for CI, participants suggested that CI could improve the on‐going care by ensuring patients are educated about what to do in case of a flare. Participants also recommended that access to publicly funded nonphysician specialists with RA expertise (eg, advanced practice nurses, physiotherapists and pharmacists), as well as the ability to contact them directly, would improve patient‐centredness of care and could be organized through CI.

Moreover, participants suggested that CI could address challenges to navigating the health‐care system not related to access to care. For example, CI may help patients cope with the unpredictable course of their disease and lack of information about RA by providing patients with education about RA, available peer support and trustworthy online resources as soon as the referral was received and/or diagnosis established.

### 3.3 Application of the PaCERs study findings in the project

During the next steps of the project, findings of the PaCERs study were used to inform the development of an evaluation framework for CI (key performance indicators [KPIs] for measuring the quality of care and a patient experience survey; Table 4) and the choice of candidate CI strategies (ie, its potential configurations) to be evaluated.

**Table 4:** Key performance indicators (KPIs)^25^ and statements in the patient experience survey aligned with the corresponding themes identified through the patient and community engagement research (PaCERs) study

Themes identified during the PaCERs study	KPIs^a^	Statements in the patient experience survey
1. Initial access to rheumatology care	KPI 2: Time from RA referral receipt to referral completion for initially incomplete referrals KPI 6: Waiting times for rheumatologist consultation for patients with new‐onset rheumatoid arthritis KPI 7: Time to disease‐modifying antirheumatic drug therapy for patients with new‐onset RA KPI 8: Percentage of patients with new‐onset RA with at least one visit to a rheumatologist in the first year of diagnosis KPI 23: Patient experience with centralized intake	Care for my rheumatoid arthritis started quickly after the referral to the rheumatology clinic The referral from my family doctor to the rheumatology clinic was dealt with in a timely manner It was difficult to reach the care providers at the rheumatology clinic
2. On‐going access to rheumatology care	KPI 17: Waiting times for patients with established RA conditions KPI 18: Percentage of patients living with RA treated with a disease‐modifying antirheumatic drug during the measurement year KPI 23: Patient experience with centralized intake	The care providers at the rheumatology clinic explained to me what to do if my rheumatoid arthritis gets worse
3. Information about RA and resources for those living with RA	KPI 11: Percentage of patients who receive information regarding resources and tools available for management while waiting for first musculoskeletal specialty contact KPI 23: Patient experience with centralized intake	The care providers at the rheumatology clinic responded to all my questions or concerns in a way I could understand I received information on other options to manage my rheumatoid arthritis (eg, physiotherapy, acupuncture, chiropractor, nonmedical wellness strategies) The care providers at the rheumatology clinic gave me information on how to self‐manage my rheumatoid arthritis The information I received on peer support groups for rheumatoid arthritis was useful
4. Fear of the future	KPI 23: Patient experience with centralized intake	The care providers at the rheumatology clinic explained the proposed treatment plan to me in a way I could understand Before my treatment for rheumatoid arthritis, all the risks and/or benefits were explained to me in a way I could understand The care providers at the rheumatology clinic explained the reasons for all the tests in a way I could understand The care providers at the rheumatology clinic explained my test results to me in a way I could understand The purpose of the medications that were prescribed for rheumatoid arthritis was explained to me in a way I could understand The information I received about rheumatoid arthritis was clear
5. Collaborative and continuous care	KPI 23: Patient experience with centralized intake	The care providers at the rheumatology clinic knew important information about my medical history My family doctor is informed and up‐to‐date about the care I receive at the rheumatology clinic My care was well‐coordinated among different care providers at the rheumatology clinic I received consistent messages from all of the different care providers at the rheumatology clinic

a The KPIs in the table refer to the numbering in the manuscript describing the process of the development of KPIs.^25^

#### 3.3.1 KPIs

The set of KPIs (ie, quantifiable measures of quality of care) to evaluate CI was developed through a multistep process described in detail elsewhere.^25^ Out of the final set of KPIs,^25^ four KPIs addressed the identified during the PaCERs study theme of “initial access to rheumatology care,” two—the theme of “on‐going access to rheumatology care,” one—the theme of “lack of information about RA and resources for those living with RA,” and another was focused on patient experience with CI in general (Table 3).

The project then used a multicriteria decision analysis (MCDA) process^26^ to develop an aggregate performance measure for CI.^27^ In the MCDA process, the KPIs identified that aligned with the themes identified during the PaCERs study were found to be of most importance (ie, were ranked higher) by all stakeholders (patients and PaCERs [n = 2], health‐care professionals [n = 10], health‐care administrators [n = 7] and academic researchers [n = 9]).^27^ The KPI focused on patient experience with CI was ranked as the top KPI.^27^ As part of the project, KPIs were developed to evaluate the quality of care delivered by an existing centralized intake and triage in rheumatology system in Calgary, Alberta, and to identify the system's gaps.^28^ Next, KPIs will be used within the implemented province‐wide redesigned CI to measure the impact of the change on quality of care.

#### 3.3.2 Patient experience survey

To address and measure the KPI about patient experience, the team developed a patient experience survey.^29^ The survey development involved discussions between PaCERs (n = 2) and academic researchers (n = 4) over the course of several meetings. During discussions, researchers built on findings from the previous activities within the project to adapt questions from several validated instruments on patients’ experience (eg Consumer Assessment of Healthcare Providers and Systems (CAHPS^®^) patient survey questions,^30^ satisfaction questionnaire for patients with RA^31^).^29^ This process resulted in the selection of a set of 23 questions (Appendix 1). The patient experience survey will be administered to patients with suspected RA referred to rheumatology clinics through the current centralized intake and triage in rheumatology system in Calgary, Alberta.^28^ These data will be compared to the patients’ experience after the system optimization.

#### 3.3.3 Candidate strategies for CI

A set of candidate strategies for optimization of CI was identified during a 1‐day stakeholder meeting. Participants (patients and PaCERs [n = 2], health‐care professionals [n = 10], health‐care administrators [n = 7] and academic researchers [n = 9]) were presented with the elements of predefined based on the literature and discussions with relevant stakeholders^32^, ^33^ candidate strategies for CI. Afterwards, participants reflected on proposed models and provided feedback to develop the preferred model for Alberta. Throughout this discussion, proposed alternative configurations of CI were refined to ensure their alignment with the previously established set of principles for an optimal, CI identified challenges to accessing and navigating the system, and the key elements of the system that would be responsive to patients’ needs. All discussion points were recorded by a scribe. The meeting notes were reviewed and analysed to select the final set of models to be evaluated.

### 3.4 Future plans

Once the candidate strategies are selected, they will be tested using simulation models^34^ delineating the operational features and describing the clinical pathway and the flow of patients. Each strategy will be tested for its ability to adjust for variation in the type of treatment needed by patients and the availability of various care providers and facilities to provide the different services to identify the strategy that would be most efficient and effective in directing patients to appropriate care providers, thus achieving improved patient and system outcomes (ie, optimal strategy). Finally, based on the feasibility and readiness of the clinics, there will be an opportunity to implement and evaluate the identified optimal strategy.

## 4 DISCUSSION

This case study adds information to the scarce body of literature on examples of patient engagement in health‐care system design. In the described case, patient engagement in the redesign of CI was fostered through the continuous engagement of patients in research with the applied focus on optimization of care delivery. The described approach to patient engagement in system redesign has a unique feature, the active engagement of PaCERs, patients trained to design and conduct health research. Throughout the project, PaCERs served as a “bridge” between patients and other stakeholders, ensuring that the patients’ voice was heard and considered during each step of the project. We believe this feature has ensured that patients’ needs and preferences were incorporated into the system redesign rather than being included in a research process as a “token patient.” This case study did not aim to assess the effectiveness of the applied approach to patient engagement in system redesign. Nonetheless, the alignment of the majority of the elements of an evaluation framework for the future CI with the key themes identified during the PaCERs study suggests that the developed framework was indeed patient‐centred. This, in turn, suggests that the applied approach to patient engagement has served as an effective tool for designing a patient‐centred system.

Despite its unique feature, the active engagement of PaCERs, our approach to patient engagement in system redesign correlates with several frameworks for understanding and classifying patient engagement discussed in the literature. For instance, a framework for patient and family engagement in health and health care by Carman et al^35^ describes engagement activities along a continuum with consultation being at the lower end and partnership and shared leadership representing the higher end of this continuum. The framework also classifies engagement based on the level at which it occurs, including direct care, organizational design and governance, and policymaking.^35^ According to this framework, our approach covers the higher end of the continuum of engagement within the level of organizational design and governance.^35^ Next, according to the framework for patient and service user engagement in research,^16^ our approach includes all components of patient and public involvement in research, such as patient and service user initiation, building reciprocal relationships, colearning process and re‐assessment and feedback, throughout both preparatory, execution and translational phases of the project. Limited feasibility of approaches that cover the higher end of the continuum of patient engagement and/or include all components of patient and public involvement in research has been discussed as a barrier to their application in practice.^16^, ^35^ The current manuscript presents an example of a successful application of such an approach.

Our findings should be considered within the context of the single‐case study design and qualitative analysis. First, the discussed approach to the patient engagement in system redesign has emerged from a unique single case, which limits the generalizability of our findings.^19^ This case study represents one approach to patient engagement in system redesign, which may not fit every question, system settings and clinical area. Nonetheless, the holistic approach of the single‐case study design facilitated an in‐depth description of the case and its context, allowing the reader to make conclusions regarding the feasibility of the approach in the local environment.^19^ Second, the elements of the system that would be responsive to the needs of patients with RA, as well as the suggestions as to how CI can help to meet those needs, were developed through a qualitative PaCERs study with a relatively homogenous group of patients with RA. To reduce the potential for selection, coding and interpretation biases,^36^ all steps of the PaCERs study were conducted using sound methodology by trained patient engagement researchers experienced in qualitative research, and data were collected until saturation was reached. All coding, content analysis and interpretation were done by at least two PaCERs, reviewed by the team and finalized when the consensus was achieved. Lastly, although our findings on challenges experienced by patients with RA align with the published literature,^28^, ^37^, ^38^, ^39^, ^40^, ^41^ the identified key elements of the system that would be responsive to needs of patients with RA, and suggestions as to how CI can help to meet those needs, might be specific to a universal system with a single public payer as in Alberta, Canada. Therefore, these key elements should be carefully considered within specific local environment.

## 5 CONCLUSIONS

Overall, this study presents a feasible multistep approach to patient engagement in health‐care system redesign. This manuscript contributes towards the understanding of the complex phenomenon of patient engagement and serves as one more step towards a patient‐centred system that is redesigned with active patient engagement.

## CONFLICT OF INTEREST

The authors have no conflicts of interest. Elena Lopatina is funded by Mitacs and Alberta Bone and Joint Health Institute through Mitacs Accelerate Internship; Deborah Marshall is supported by a Canada Research Chair, Health Systems and Services Research, and the Arthur J.E. Child Chair Rheumatology Outcomes Research.

## AUTHORS’ CONTRIBUTIONS

All authors meet the authorship criteria of the International Committee of Medical Journal Editors: Elena Lopatina, Jean L. Miller, Sylvia R. Teare, Nancy J. Marlett, Jatin Patel, Claire E.H. Barber, Dianne P. Mosher, Tracy Wasylak, Linda J. Woodhouse and Deborah A. Marshall provided substantial contribution to conception and design of the study, as well as analysis and interpretation of data, participated in drafting and revising the article, and provided final approval on the version to be submitted; Deborah Marshall as a corresponding author will act as the overall guarantor.

## Appendix 1 Patient experience survey [1]

### 1.1

**Table:**

**Partnership for Research and Innovation in the Health System (PRIHS) grant: Optimizing centralized intake to improve arthritis care for Albertans**		

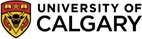
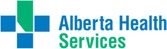
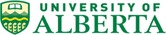
		

### Rheumatology Clinic Patient Experience Survey

#### What is the survey about?

This survey is about your experience as a rheumatology patient in our clinic.

#### Who should complete the survey?

The survey should be completed by rheumatology patients who receive their care at the University of Calgary rheumatology clinics at either the Richmond Road Diagnostic and Treatment Center or the South Health Campus.

#### How to complete the survey?

Please select only one answer that shows how much you agree with the following statements.

**Table:**

Statement	Strongly Agree	Agree	Disagree	Strongly Disagree	Not Applicable
1. My care started quickly after the referral to the rheumatology patient clinic	□	□	□	□	□
2. The referral from my family doctor to the rheumatology patient clinic was dealt with in a timely manner	□	□	□	□	□
3. It was difficult to reach the care providers at the rheumatology patient clinic	□	□	□	□	□
4. The care providers at the rheumatology patient clinic knew important information about my medical history	□	□	□	□	□
5. My family doctor is informed and up‐to‐date about the care I receive at the rheumatology patient clinic	□	□	□	□	□
6. My care was well‐coordinated among different care providers at the rheumatology patient clinic	□	□	□	□	□
7. I received consistent messages from all of the different care providers at the rheumatology patient clinic	□	□	□	□	□
8. The care providers at the rheumatology patient clinic respected my wishes and ideas about my treatment	□	□	□	□	□
9. I was as involved as I wanted to be in making decisions about my treatment	□	□	□	□	□
10. The care providers at the rheumatology patient clinic asked me about my goals for treatment and what is important to me in managing my condition	□	□	□	□	□
11. The care providers at the rheumatology patient clinic responded to all my questions or concerns in a way I could understand	□	□	□	□	□
12. The care providers at the rheumatology patient clinic explained the proposed treatment plan to me in a way I could understand	□	□	□	□	□
13. Before my treatment, all the risks and/or benefits were explained to me in a way I could understand	□	□	□	□	□
14. The care providers at the rheumatology patient clinic explained the reasons for all the tests in a way I could understand	□	□	□	□	□
15. The care providers at the rheumatology patient clinic explained my test results to me in a way I could understand	□	□	□	□	□
16. The purpose of the medications that were prescribed were explained to me in a way I could understand	□	□	□	□	□
17. The information I received about my condition was clear	□	□	□	□	□
18. I received information on other options to manage my condition (eg, physiotherapy, acupuncture, chiropractor, nonmedical wellness strategies)	□	□	□	□	□
19. The care providers at the rheumatology patient clinic gave me information on how to self‐manage	□	□	□	□	□
20. The care providers at the rheumatology patient clinic explained to me what to do if my rheumatoid arthritis gets worse	□	□	□	□	□
21. The information I received on peer support groups was useful	□	□	□	□	□
22. Overall, I was treated with respect while I was at the rheumatology patient clinic	□	□	□	□	□
23. The care providers at the clinic made efforts to understand what having arthritis means to me	□	□	□	□	□
**Additional comments**					
__________________________________________________________________________________________________________________________________________________________________________________________________________________________________________________________________________________________________________________________________________________________________________________________________________________________________________________________________					

**Thank you for your participation**

Source: Carr et al,^29^
